# Biomimetic Antibacterial Pro-Osteogenic Cu-Sericin MOFs for Osteomyelitis Treatment

**DOI:** 10.3390/biomimetics7020064

**Published:** 2022-05-20

**Authors:** Banani Kundu, Rui L. Reis, Subhas C. Kundu

**Affiliations:** 1 3B’s Research Group, I3Bs—Research Institute on Biomaterials, Biodegradables and Biomimetics, University of Minho, Headquarters of the European Institute of Excellence on Tissue Engineering and Regenerative Medicine, AvePark, Parque de Ciência e Tecnologia, Zona Industrial da Gandra, 4805-017 Barco, Portugal; rgreis@i3bs.uminho.pt (R.L.R.); kundu@i3bs.uminho.pt (S.C.K.); 2 ICVS/3B’s—PT Government Associate Laboratory, 4710-057 Braga, Portugal

**Keywords:** copper, silk sericin, metal–organic framework, antibacterial activity, osteogenesis

## Abstract

Osteomyelitis is an inflammation of the bone caused by bacterial infection. It usually develops from broken bones, decayed teeth, or heavily punctured wounds. Multi-drug-resistant bacteria are the major hurdle in the treatment of osteomyelitis. The ever-rising antibiotic resistance even leads to amputations or fatalities as a consequence of chronic osteomyelitis. Hence, a single agent with antibacterial activity as well as bone regenerative properties can serve as a potential off-the-shelf product in the treatment of osteomyelitis. Herein, the antibacterial and pro-osteogenic characteristics of copper sericin (Cu-SER) metal–organic frameworks (MOFs) are reported. Sericin, a silk protein with antibacterial activity and an osteoinduction property, acts as an organic template for the deposition of Cu-SER MOFs, similar to collagen during biomineralization in bone. The MOFs exhibit cytocompatibility and osteogenic activity in a dose-dependent manner, as revealed by cell proliferation (alamarBlue) and mineralization (Alizarin Red S and Energy Dispersive X-ray analysis). The bactericidal activity of Cu-SER MOFs was investigated by scanning electron microscopy and a growth kinetic analysis. Together, the report illuminates the unique phenomenon of Cu-SER MOFs that kill bacteria upon contact while being well-tolerated by primary human cells. Hence, Cu-SER MOFs hold the potential to minimize antibiotic dependence.

## 1. Introduction

The global osteomyelitis market is estimated to be worth USD (United States dollar) 1.64 billion in 2021, with a projected value of USD 2.5 billion from 2022 to 2027 [1]. The key pathogens responsible for osteomyelitis are *Staphylococcus aureus* and *Escherichia coli* as well as multidrug-resistant bacteria such as *Pseudomonas aeruginosa* and *Acinetobacter baumannii* [2]. Though Gram-positive bacteria cause the severe infection in the initial phase, the relapse infection involves both Gram-positive and Gram-negative bacteria, including methicillin-sensitive *S. aureus*. The site of bone infection exhibits poor vascularity, hence, often requires prolonged and high concentrations of antibiotics, which leads to antibacterial resistance.

Antimicrobial resistance (AMR) contributes to approximately 7 million deaths per year globally. If current trends continue, AMR will kill 10 million people per year by 2050, which is more than cancer [3]. Due to the overuse or misuse of antibiotics in patients during a pandemic, confronting AMR will be the most critical challenge in healthcare post-COVID-19. It has been 30 years since the development of the last antibiotic agent, and just 3 out of a total of 41 recently developed antibiotics are currently sensitive against resistant bacteria [4]. Even the new antibiotics that are going to be on the market shortly are only temporary options before resistance develops. Moreover, the high production cost of these antibiotics limits their availability in low-resource countries. Hence, it is crucial to identify alternative low-cost antimicrobial agents that can treat AMR infections. The repurposing of available potential MOFs to address the current shortage of antimicrobial agents can be a useful and life-saving strategy. Hence, this report repurposes the super-adsorbent (readily adsorbs heavy metal ions of Pb (II), Cd (II), and Hg (II)) copper (Cu)–silk sericin (SER) MOFs [5] for the treatment of osteomyelitis.

Recent research interest in antimicrobial agents focuses on Cu due to its biocidal effect against a wide range of pathogens, including bacteria, fungi, and viruses [6], that even extends to methicillin-resistant *Staphylococcus aureus* (MRSA) [7] and vancomycin-resistant *Enterococcus* [1]. Acknowledging the high therapeutic potential of copper, Cu-based products have been approved by the US Environmental Protection Agency since February 2008 for human use [8]. However, in the design of therapeutics, minimal cytotoxicity towards healthy cells is desired. This is achieved by linking copper with biocompatible organic templates, such as proteins, via coordination bonds [9].

Silk protein sericin (SER) possesses antibacterial activity [10]. Diverse metal ions have successfully been complexed with SER to further improve the antibacterial properties of the resultant biomaterials [11] or textiles [12]. SER is a highly hydrophilic glue-like glycoprotein with high serine and threonine contents [13]. SER serves as a template for the biomimetic nucleation of hydroxyapatites and promotes the osteogenic differentiation of human bone-marrow-derived stem cells [14]. SER increases the expression of bone morphogenic protein-2/4, which in turn facilitates osteogenesis in bone defects [15]. The secondary metabolites of SER are polyphenols and flavonoids that act as pro-oxidants, elevate the intracellular ROS, and cause the selective death of cancer cells at high concentrations without affecting healthy cells [16]. SER is a byproduct of the silk-based textile industry, and the addition of it to industrial waste increases the global burden of pollution. The recovery cost of SER from wastewater is very cheap. Hence, the recovery of SER from wastewater and using it to develop commercial healthcare products of need is not only economical but also eco-friendly, contributing to global healthcare sustainability.

Herein, the present study describes the one-pot facile fabrication of Cu-silk sericin metal–organic frameworks, where amino acids act as green reductants for the nucleation of copper phosphate crystals. The comprehensive overview of its in vitro antibacterial activity is analyzed and discussed. In contrast to water, the interaction of particles within a cell or biological fluid is not only limited to electrostatic interaction, but other forces, such as hydrophilic–hydrophobic and Van der Waal’s interactions, play a critical role, resulting in an interfacial potential, which is taken into consideration in the present study to understand its antibacterial activity. To couple its antibacterial activity with its cytocompatibility and osteo-induction ability, human adipose stem cells are used in the present investigation.

## 2. Materials and Methods

### 2.1. Preparation of Silk Sericin

Sericin was isolated from *Bombyx mori* silk cocoons using sodium carbonate (0.02 M, Sigma-Aldrich, St. Louis, MO, USA) [17]. Desirable concentrations of silk sericin (5 mg/mL) were achieved by further dialyzing the protein solution against a 30% (*w*/*v*) polyethylene glycol (6000 g/mol) solution at room temperature (dialysis membrane, MWCO 3500) [13]. The dilutions of silk sericin were made using deionized water.

### 2.2. Synthesis of Copper-Silk Sericin MOFs

The Cu-SER MOFs were obtained using the standard protocol with slight modifications [5]. In brief, an appropriate amount of aqueous CuSO_4_ (Sigma-Aldrich, St. Louis, MO, USA) solution (1 mM in the final reaction mixture) was added to Dulbecco’s phosphate-buffered saline (1 × DPBS, pH 7.4) containing silk sericin (1–5 mg/mL). The reaction mixture was mixed gently and left undisturbed for 8 h at room temperature. A sky-blue-colored precipitation of Cu-sericin MOFs (Cu-SER) was obtained by centrifugation and washed repeatedly with deionized water. The Cu-SER MOFs were then frozen overnight at −20 °C, followed by freeze-drying to obtain the Cu-SER MOF powders. The Cu-SER MOFs were termed SER1-SER5 based on the initial concentration of SER solution involved in fabrication.

### 2.3. Biophysical Characterization

The microarchitecture of Cu-SER was observed after being sputter-coated with platinum (5 nm) and imaged using a scanning electron microscope (JEOL JSM 6301F/Oxford INCA Energy 350/Gatan Alto 2500, Tokyo, Japan). The images were further processed using ImageJ (National Institutes of Health, version 1.5. For elemental analysis, the Cu-SER MOFs were scanned using a scanning electron microscope (JEOL JSM 6301F/Oxford INCA Energy 350/Gatan Alto 2500, Tokyo, Japan) coupled with energy-dispersive X-ray spectroscopy (EDS) (JEOL JSM, Tokyo, Japan).

### 2.4. Isolation and Culture of Cells

Human adipose-derived stem cells (hASCs) were obtained from Hospital da Prelada (Porto, Portugal) with the informed consent of patients after liposuction and were processed at 3B’s Research Group, Minho University, Portugal following the institutional protocol [18]. The cells were cultured in αMEM (Life Technologies, Renfrew, UK) supplemented with 10% *v*/*v* fetal bovine serum (FBS) and 1% *v*/*v* penicillin/streptomycin (PS) at 37 °C in a 5% CO_2_ humidified atmosphere. hASCs from passage three were used for the experiments.

Once confluency was reached, the hASCs were trypsinized, brought into suspension, and seeded in 12-well plates at a concentration of 5 × 10^4^ cells/well. The cells were allowed to adhere overnight at 37 °C in a 5% CO_2_ atmosphere. Next, the adhered monolayer of hASCs was subjected to different dilutions of Cu-SER (500–62.5 μg/mL prepared in αMEM-10% *v*/*v* FBS-1% *v*/*v* PS) for a week with a replacement of media every other day. The experiment was run in triplicate for each dilution. As a control, hASCs were cultured in osteogenic medium only (MEM supplemented with 10 mM glycerol phosphate, 10 mM dexamethasone, and 50 μg/mL ascorbic acid) [19].

### 2.5. alamarBlue Assay

The alamarBlue assay (Bio-Rad, Watford, UK) was used to assess cell proliferation after treatment with various dilutions of Cu-SER MOFs (500–62.5 g/mL). At each pre-selected time point (day 1 and 7), the culture medium was aspirated from the wells, and the cells were incubated with freshly prepared alamarBlue (10% *v*/*v*, diluted in basal culture media) for 4 h at 37 °C and 5% CO_2_. The fluorescence of the alamarBlue solution was measured using a spectrofluorometer (Synergy HT, Bio-Tek, Winooski, VT, USA) at excitation and emission wavelengths of 530 and 590 nm, respectively.

### 2.6. Fluorescence Microscopy

At the end of the culture (day 7), the cell monolayers (treated with a Cu-SER MOF dilution of 62.5 g/mL) were rinsed with PBS and fixed in 10% formalin (ENZIfarma, Lisbon, Portugal) for 30 min at room temperature. Following fixation, the monolayers were gently rinsed with PBS (pH 7.4) and stained with rhodamine-labeled phalloidin (1:500 *v/v* in PBS, Sigma-Aldrich, St. Louis, MO, USA), counterstained with 4′, 6-diamidi-no-2-phenylindole (DAPI, 1:1000 *v/v* in PBS, Sigma-Aldrich, St. Louis, MO, USA) for nuclei. After staining, the arrangement of actin filaments was visualized using an Axioplan Imager Z1 fluorescence microscope (Zeiss, Jena, Germany).

### 2.7. Histology Analysis

For mineralization, the cells were cultured for 14 days (with a Cu-SER MOF dilution of 62.5 g/mL) and fixed as mentioned above (Section 2.6). Post-fixation, the deposition of calcium was investigated by Alizarin Red S staining [20]. The cell monolayers were incubated with the Alizarin Red S staining solution (vWR, Leicestershire, UK) for 30 min at room temperature, followed by washing with PBS (pH 7.4) to eliminate extra dye. The cells were imaged using a Leica DM750 (Jena, Germany) with an MRc5 camera.

### 2.8. Antibacterial Activity

*Staphylococcus aureus* (American Type Culture Collection, ATCC 25923) and *Escherichia coli* (*E. coli* ATCC 25922) cultures were prepared by inoculating a single bacterial colony into respective nutrient broths and incubating at 37 °C under constant shaking (150 rpm).

For surface potential measurement using a Zetasizer (Malvern, Worcestershire, UK), bacterial cultures, after overnight incubation, were harvested by centrifugation (5000 rpm, 10 min at 4 °C), followed by washing using HEPES buffer (10 mM, pH 7.4) (Gibco^®^, UK) and were resuspended in HEPES buffer. Dilutions of Cu-SER MOFs (500–125 μg/mL) were also prepared in HEPES buffer. For bacteria interfacial potential measurement, approximately 100 μL of Cu-SER dilution was added into 900 μL of bacterial culture broth to obtain a final 1 × 10^5^ CFU/mL and it was incubated for 1 h at room temperature before surface potential measurement. Bacterial cells prepared in HEPES buffer to the same dilution without any MOF treatment and incubated for 1 h at room temperature served as controls.

A 500 μg/mL dilution of Cu-SER MOFs was used to treat 10^7^ CFU/mL of log phase *S. aureus* and *E. coli* at 37 °C. The growth curve over time (24 h) was plotted in reference to the optical density (OD at 600 nm) [20]. To visualize the morphology of bacteria after treatment, the bacterial pellet was collected upon centrifugation (6000× *g* for 8 min), washed with PBS (pH 7.4), and fixed with 10% (*v*/*v*) formaldehyde. A drop of the sample was then placed on the cover slip, dried under a sterile hood, and imaged using the JEOL JSM 6301F/Oxford INCA Energy 350/Gatan Alto 2500.

### 2.9. Statistical Analysis

The statistical analysis was carried out using a one-way analysis of variance (ANOVA) and the results were presented as means ± standard deviations (*n* = 3). Statistical significance was considered at *p* < 0.1.

## 3. Results

### 3.1. Formation of Cu-SER MOFs

**Figure 1:**
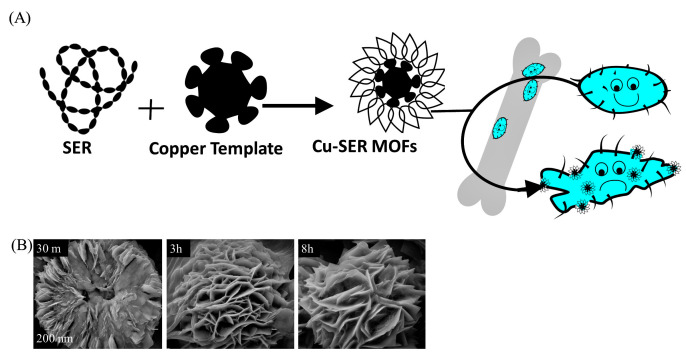
(**A**) Illustration of the self-assembly of Cu-SER MOFs and schematic of the investigation. (**B**) SEM images representing the time-dependent growth of MOFs (1 mg/mL SER, 1 mM CuSO_4_): 30 min, 3 h, and 8 h of incubation time.

A schematic depiction of the preparation of hybrid metal–organic frameworks (MOFs) is summarized in Figure 1A. By adding CuSO_4_ to SER prepared in phosphate buffer, a light blue precipitation of Cu-SER MOFs appeared. Figure 1B represents the scanning electron micrographs (SEM) of the time-dependent development of Cu-SER MOFs. The effect of the reaction duration on MOF growth (with 1 mg/mL SER and 1 mM CuSO_4_) revealed that the kinetically controlled crystal growth of copper phosphate achieved stability and a flower-like structure after 8 h (Figure 1B). In this proposed process of growth, the crystal generation initiates at each Cu^2+^ binding site of the agglomerates, leading to the appearance of individual petals. Finally, the anisotropic growth ends in the formation of a completely branched flower-like structure.

**Figure 2:**
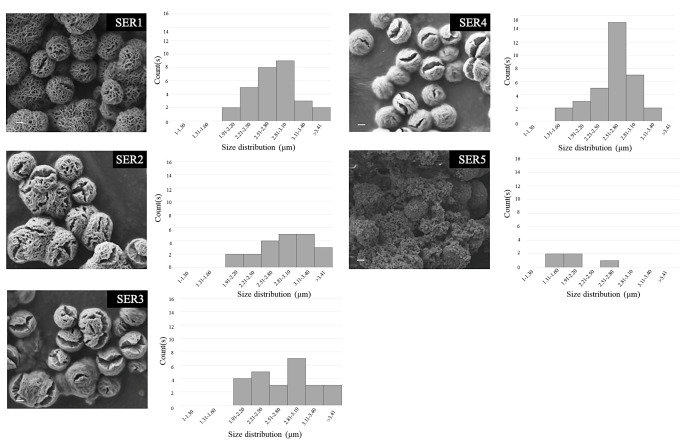
Representative scanning electron microscopy images of Cu-SER MOFs obtained using different silk sericin concentrations (1–5 mg/mL) and their size distribution.

**Figure 3:**
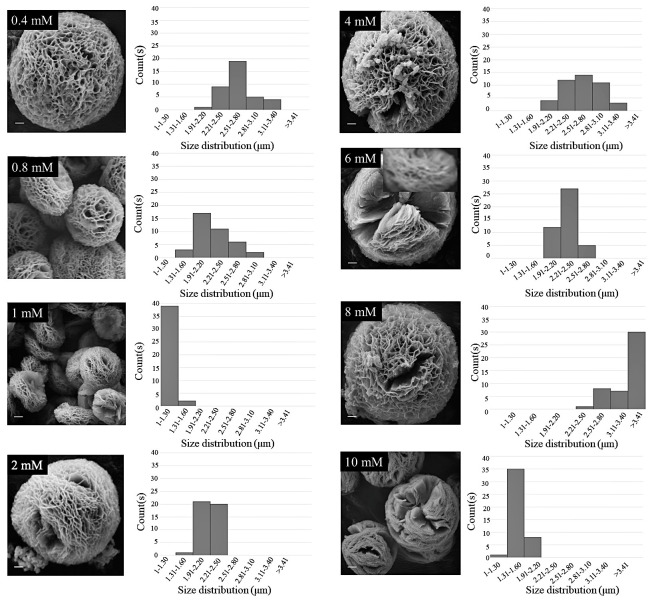
SEM images of Cu-SER MOFs exhibiting different morphologies at different Cu^2+^ concentrations. Scale bar = 100 nm (0.8 mM, 1 mM), 200 nm (2 mM, 4 mM, 6 mM, 8 mM), and 300 nm (0.4 mM, 10 mM).

SEM images (Figure 2) are suggestive of a concentration-dependent self-assembly of silk SER using an inorganic Cu template. The SER protein molecules formed complexes with Cu^2+^ by using the co-ordination facility of amide groups in the protein backbone. Along with the amide backbone, the polar side chains, such as carboxyl, hydroxyl, and amine groups, also interacted with Cu^2+^, facilitating the generation of folding in the petals. The crystals of copper phosphate generated from individual Cu^2+^ binding sites resulted in separate petals. In this crystal growth process, silk SER served as the glue to hold the petals together, forming a scaffold-like structure. With the decreasing protein concentration, there was a reduction in nucleation sites, leading to a looser structure (Figure 2). To optimize the reaction conditions, a systematic investigation was carried out, varying the concentration of CuSO_4_ (Figure 3), which revealed that the architecture of as-grown biomaterials was critically regulated by the concentration of precursor molecules (both CuSO_4_ and silk protein).

A mixture of 1 mg/mL SER and CuSO_4_ concentrations ranging from 0.4 mM to 10 mM in 1 X DPBS regulated a variety of hybrid structures, some with ambiguous morphologies (>8 mM) (Figure 3). A closer look at the petals, such as the inset of 6 mM CuSO_4_ (Figure 3), revealed a multi-layer porous morphology formed by the combination of several nano-sheets.

Among the tested CuSO_4_ concentrations (0.4 mM to 10 mM), the 1 mM concentration produced the fewest (1.1–1.2 m) and most homogeneously distributed Cu-SER MOFs (Figure 3). Since the aim of the present investigation was to augment the antibacterial and osteogenic activity of Cu-MOFs by using silk SER, further experiments were continued using only CuSO_4_ (1 mM) and SER (1–5 mg/mL), designated as SER1–SER5, respectively.

### 3.2. Pro-Osteogenic Activity of Cu-SER MOFs

**Figure 4:**
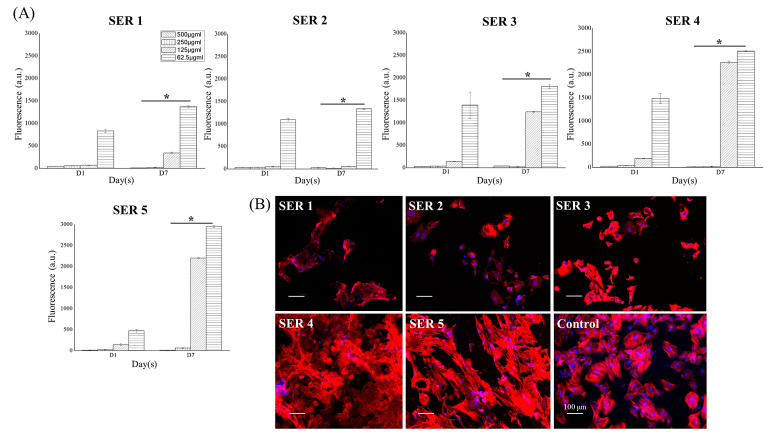
(**A**) Concentration-dependent viability of hASCs after treatment with different dilutions of Cu-SER MOFs. * *p* < 0.1. (**B**) Cytoskeleton staining of hASCs after treatment with MOFs for 7 days. Actin filaments are stained with phalloidin (red) and nuclei are in blue. Scale bar = 100 μm.

Human adipose-derived stem cells (hASCs) were viable and proliferative in 7 days of culture with diverse concentrations of Cu-SER MOFs (500–62.5 μg/mL) in a dose and SER-concentration-dependent manner (Figure 4A). The highest cell viability was observed at 62.5 μg/mL. The viability of cells was reduced as the concentration of MOFs increased. Due to this, further cell culture experiments were continued using 62.5 μg/mL only. The cytoskeleton arrangement is a biomarker of cell health and differentiation. After 7 days of culture in osteogenic media, hASCs revealed a characteristic fibroblast-like phenotype containing actin stress fibers spreading across the cytoplasm (Figure 4B, Control). In contrast, Cu-SER MOF-treated cells exhibited limitedly stretched actin filaments, except in Cu-SER5-treated cells (Figure 4B). The stress fibers in the Cu-SER5-treated hASCs were denser with crisscross patterning, which is typical of mesenchymal stem-cell-derived osteoblasts [19], indicating the shift in the phenotype of hASCs. This actin arrangement of hASCs is associated with osteogenesis [19], demonstrating the pro-osteogenic behavior of hASCs after Cu-SER MOF treatment.

**Figure 5:**
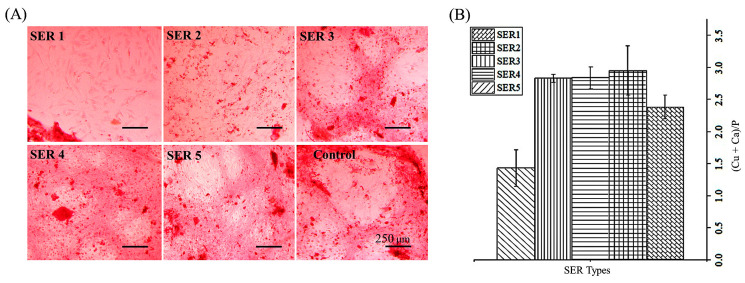
(**A**) Histological analysis (Alizarin Red S staining) of mineralization by hASCs treated with MOFs. Scale bar = 250 μm. (**B**) Atom percentage ratio of calcium and copper to phosphate in crystallites.

The osteogenic capacity of stem cells was confirmed by Ca-mineralization, which was visualized by Alizarin Red S staining. An intense colorization compared to that of control was revealed by Cu-SER5-treated cells (Figure 5A). The elemental map of the apatites obtained in Cu-SER MOFs showed a (Ca+Cu)/P = 1.4 ± 0.28 for Cu-SER5 (Figure 5B), which was close to the theoretical value for stoichiometric hydroxyapatite (1.67).

### 3.3. Antibacterial Activity 

**Figure 6:**
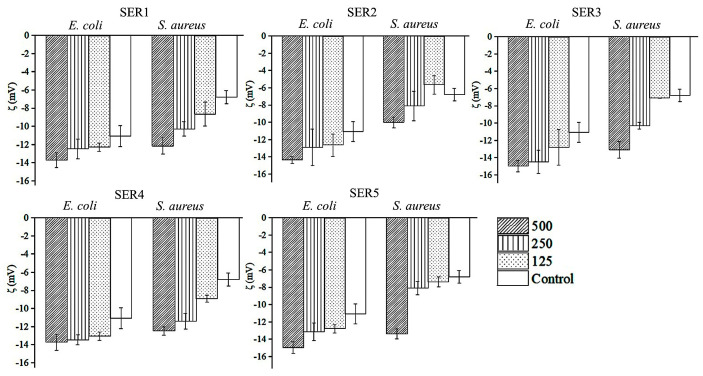
Effect of treatment of Cu-SER MOFs on bacterial cell surface zeta potential of *Staphylococcus aureus* and *Escherichia coli*.

**Figure 7:**
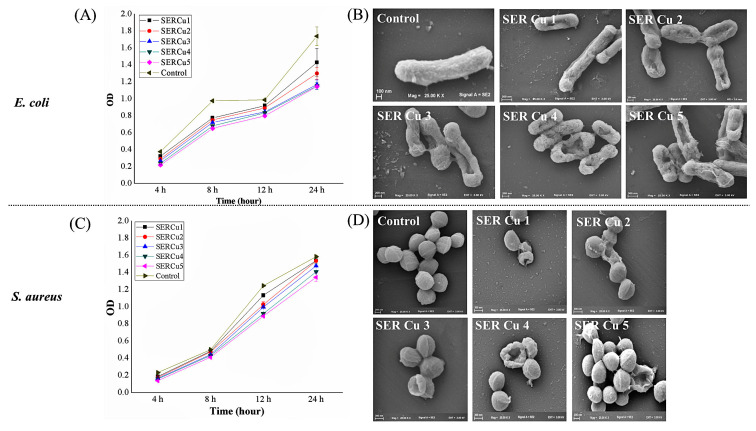
(**A**) Growth kinetics of *E. coli* treated with 500 μg/mL of Cu-SER MOFs for 24 h. (**B**) Scanning electron micrographs of *E. coli* post-treatment. Scale bar = 100 nm (Control), 200 nm (SER Cu 2 and SER Cu 4), and 300 nm (SER Cu 1 and SER Cu 5). (**C**) Growth kinetics of *S. aureus* treated with 500 μg/mL of Cu-SER MOFs for 24 h. (**D**) Visualization of bacterial cell surface by SEM. Scale bar = 100 nm (Control), 200 nm (SER Cu 2 and SER Cu 4), and 300 nm (SER Cu 1 and SER Cu 5).

The zeta potential was used to investigate how Cu-SER MOFs neutralize the surface potential of bacteria cells (Figure 6). The untreated *S. aureus* and *E. coli* demonstrated zeta potentials of −6.7 mV and −11.05 mV, respectively. The zeta measurement of MOF-treated samples exerted significant variations in interfacial potential for both bacteria, though not to the same extent. This change in surface potential (at 500 μg/mL concentration) is adequate to destabilize the bacterial membrane, which in turn affects the viability of bacteria. As a result, further antibacterial activity was investigated using this concentration only. Growth kinetic studies in the presence and absence of Cu-SER MOFs revealed that lower sericin concentrations in MOFs (1–3 mg/mL) inhibited *E. coli* more than *S. aureus* (Figure 7A,C). The SEM images represented the membrane deformity (Figure 7B,D).

## 4. Discussion

The ideal agent for the treatment of osteomyelitis must not only have resistance to bacterial infection but also have the capability to stimulate cellular proliferation and osteo-differentiation. Currently, the concept of green chemistry, as well as concerns about the long-term production of environmental friendly antimicrobial agents, is driving the research into natural antimicrobial biomaterials, such as silk sericin [13].

The latest advances in copper (Cu)-based biomaterials with osteogenic and antibacterial properties in bone tissue engineering have been illustrated well by Shen et al. [21]. According to the comprehensive review, adding Cu to complex hydroxyapatite-containing materials results in better bone substitutes. Stimulated body fluid (SBF) is profoundly used to induce biomimetic apatite formation [22]. However, the use of SBF solution is challenging due to the instability of the solution, prolonged nucleation time (at least 24 h), and critical preparation steps. Dulbecco’s phosphate-buffered saline (DPBS) has recently been proposed as an alternative to SBF [23].

The addition of Cu^2+^ ions (of CuSO_4_) into SER in DPBS allows the chelation of Cu^2+^ with the amino and carboxyl groups of SER [24], leading to the formation of self-assembled particular MOFs and the precipitation of copper phosphate [5]. With an increase in protein concentration, there is a local collapse and condensation of SER chains, which results in a dense hierarchical architecture [24].

Despite the importance of Cu in osteoblastic proliferation [25] and the proposed role of copper peptides in the regeneration of vascularized bone in critical-sized cranial defects of Sprague Dawley rats [26], very little is known about their direct role in osteointegration. The Ca:P ratio of 1.67 is associated with the stoichiometric hydroxyapatite and is of particular interest due to its inductive role in bone regeneration [19]. In the present report, the (Ca+Cu):P is proposed to be interesting due to the observed differentiation of hASCs. The Cu-SER5 MOFs represent the (Ca+Cu):P ratio of 1.4, in which the Ca of apatite is hypothesized to be replaced by divalent Cu (II) [27]. Interestingly, the non-stoichiometric hydroxyapatite is an essential constituent of hard tissues, such as bones and teeth [28]. Thus, the present report suggests that (Ca+Cu):P would serve as a useful indicator of the osteogenic properties of potential MOFs. The actin arrangements and presence of Ca nodule do not preclude the possible existence of some useful relationship between (Ca+Cu):P and osteoinduction, but further investigation, including an analysis of the genes and secretome, is warranted for this specific claim.

The mode of antibacterial activity of Cu is multifaceted. Either the released Cu ions impart the cytotoxicity [29] or the accumulation and dissolution in the bacterial membrane results in a change in the surface potential and permeability, with the succeeding release of intracellular biomolecules [30]. The higher concentration of Cu-SER MOFs resulted in the interfacial potential at the MOF–bacterial interface to become neutral, leading to the neutralization and release of energy, which either involves ROS production or a change in the surface tension of bacteria or both [31], leading to bacterial non-viability. The presence of an additional layer of negatively charged lipopolysaccharide in Gram-negative bacteria compared to Gram-positive bacteria resulted in relative repulsive interactions between surfaces [32].

Surface neutralization is greatly attributed to this biological phenomenon to harmonize between negatively charged MOFs and the bacterial surface potential based on an available polar or non-polar functional group on the surface [33]. So far, the known copper-dependent enzymes of bacteria are localized outside of the cytosol, arguing that it is not essential for copper ions to enter into bacterial cells to impart its toxicity [34]. In contrast, eukaryotic cells possess copper-dependent enzymes in the cytosol (in the mitochondria or nucleus). Therefore, intracellular uptake is needed. The concentration of Cu-SER MOFs that exerts potential antibacterial activity in the present report is cytotoxic for mammalian cells. The difference in the cytotoxicity of Cu-SER MOFs between bacteria and mammalian cells is anticipated to enable a systematic study of the release of Cu ions from MOFs and its transportation across the mammalian cell membrane as well as suggest further modifications in its synthesis for the clinical application of this hybrid material.

## 5. Conclusions

In summary, the facile one-pot all-aqueous-based synthesis of inorganic–protein hybrid pro-osteogenic MOFs reported herein is cost-effective. The cytotoxicity and anti-bacterial activity of the MOFs can readily be modulated by varying the concentration of sericin in the MOFs. The interfacial potential measured using zeta potential measurement after bacterial encounters with MOFs is indicative of the generation of a surface tension, leading to high lateral stress in the bacterial cell membrane. This causes irreversible membrane damage through membrane rupture or blebbing and is revealed in SEM images. Considering the selective toxicity to bacterial cells, the great potential of SER5-MOFs in clinical applications is foreseen.

## Data Availability

Not applicable.
